# Tip60 is associated with resistance to X‐ray irradiation in prostate cancer

**DOI:** 10.1002/2211-5463.12371

**Published:** 2017-12-28

**Authors:** Xin Xie, Zhaoping Xu, Chenghe Wang, Chen Fang, Juping Zhao, Le Xu, Xiaoqiang Qian, Jun Dai, Fukang Sun, Danfeng Xu, Wei He

**Affiliations:** ^1^ Department of Urology Ruijin Hospital Shanghai Jiaotong University, School of Medicine China

**Keywords:** ataxia telangiectasia mutant, prostate cancer, radioresistance, Tip60

## Abstract

Tip60, an oncogene, accelerates cell growth by regulating androgen receptor translocation into the nucleus in prostate cancer. However, the mechanism of Tip60 in the response of prostate cancer to radiotherapy, and radioresistance, has not been studied. Using human prostate cancer samples and two human prostate cancer cell lines (LNCaP and DU145), Tip60 protein expression and the acetylation of ataxia telangiectasia mutant (ATM) were analysed by western blotting and immunoprecipitation. Tip60 was downregulated with small interfering RNA. Cells were irradiated using X‐rays at 0.25 Gy·min^−1^. Cell viability was assessed by the MTT assay. The expression of Tip60 protein was increased in radioresistant prostate cancer tissues in comparison with radiosensitive tissues, which was also confirmed in both irradiated DU145 and LNCaP cells. Furthermore, the acetylation of ATM was also upregulated in a time‐dependent manner after irradiation of both DU145 and LNCaP cells. Additionally, depletion of Tip60 decreased the survival of LNCaP and DU145 cells by inducing apoptosis, reduced the acetylation of ATM and decreased the expression of phosphorylated ATM, Chk2 and cdc25A in both DU145 and LNCaP cells after X‐ray irradiation. The results of this study demonstrated that the expression of Tip60 may be related to the radioresistance of prostate cancer and could serve as a promising predictive factor for prostate cancer patients receiving radiotherapy.

**Abbreviations**
AR: androgen receptor
ATM: ataxia telangiectasia mutant
HATs: histone acetyltransferases
siRNA: lentivirus‐mediated small interfering RNA

Prostate cancer is the second most common cancer in males worldwide 1. Currently, there are over 3 300 000 prostate cancer patients in the USA, and newly diagnosed cases are increasing 2. In China, there are 603 000 prostate cancer cases and 266 000 deaths from this disease in 2015 3. Although the 5‐year survival from prostate cancer has strikingly increased with the development of new treatment methods, it remains one of the leading causes of cancer death in men worldwide 4.

In recent years, surgery, endocrine therapy, radiotherapy and chemotherapy have been the basic therapeutic modalities used in prostate cancer. However, relapse is found in approximately 10–70% of prostate cancer patients treated with radiotherapy, indicating that some cancer cells may be resistant to this treatment 5, 6. Once recurrence or metastasis occurs in this disease, the tumours often became intractable and typical chemotherapy combined with radiotherapy does not provide effective treatment 7. Thus, ascertaining the potential functional mechanisms of prostate cancer can aid in developing novel targeted therapies for radiation‐resistant cells and improve the prognosis of patients.

Lysine acetyltransferase 5, also known as Tip60, was first reported by Kamine *et al*. 8 as a cofactor of Tat, which is a transactivator of human immunodeficiency virus gene expression. It was also reported that the Tip60 protein is an important member of the MYST family of histone acetyltransferases (HATs) 9. Subsequent studies have shown that the Tip60–HAT complex plays a critical role in DNA repair and apoptosis and that ataxia telangiectasia mutant (ATM) kinase is also activated by the binding of Tip60 to histone H3 trimethylated at lysine 9 10, 11, 12.

Through large‐scale RNA interference screening, Berns *et al*. 13 found that Tip60 was an integral part of the process of p53‐dependent cell growth arrest. Moreover, Gregoire *et al*. 14 identified Tip60 as a new regulator of the mesenchymal‐to‐epithelial transition that was linked to retaining the mesenchymal phenotype of the MDA‐MB‐231 breast cancer cell line. Additionally, Tip60 played a role as a co‐activator to interact with many steroid hormone receptors, such as the androgen receptor (AR) 15, that participate in the occurrence and development of prostate cancer. Other research indicated that Tip60 could facilitate AR acetylation to improve its transcriptional activity 16, 17, 18. Furthermore, the expression of Tip60 was increased significantly in clinical prostate cancer tissues and castration‐resistant prostate cancer cells, and its expression level was closely related with progression of the disease 19. However, the functional mechanism of Tip60 in radiation‐resistant prostate cancer has not been studied.

In this study, the Tip60 gene was depleted in the LNCaP and DU145 prostate cancer cell lines using the lentivirus‐mediated small interfering RNA (siRNA) method to analyse the potential mechanism of Tip60 in the radiation resistance of prostate cancer cells *in vitro*. The results indicate that Tip60 may be regarded as an underlying therapeutic target or a prospective prognostic marker for prostate cancer.

## Materials and methods

### Patient samples

Human prostate cancer samples, including radioresistant and radiosensitive samples, were obtained from Ruijin Hospital, Medical College, Shanghai Jiaotong University. The experiments were approved by the Ethics Committee of Ruijin Hospital, Medical College, Shanghai Jiaotong University. All patients provided written informed consent.

### Cell lines

The human embryonic kidney 293T (HEK293T) cell line and two human prostate cancer cell lines (LNCaP and DU145) were purchased from the Cell Bank of the Chinese Academy of Science (Shanghai, China). LNCaP and DU145 cells were grown in Ham's F‐12 (Gibco, Gaithersburg, MD, USA) medium containing 10% fetal bovine serum (Gibco) and 1% nonessential amino acids (HyClone, Little Chalfont, UK). HEK293T cells were cultured in Dulbecco's modified Eagle's medium (HyClone) with 10% fetal bovine serum. All cell lines were maintained at 37 °C in a humidified incubator with 5% CO_2_.

### Cell irradiation

Cells were irradiated using X‐rays at 0.25 Gy·min^−1^ by a Pantak HF 420 RX machine.

### Construction of the recombinant lentivirus and lentivirus infection

Construction of the recombinant lentivirus has been described previously 20. The short hairpin oligonucleotides (shTip60: 5′‐CCTCAATCTCATCAACTACTACTCGAGTAGTAGTTGATGAGATTGAGG‐3′ and shCon: 5′‐CAACAAGATGAAGAGCACCAACTCGAGTTGGTGCTCTTCATCTTGTTG ‐3′) were synthesized and cloned into the lentivirus‐based vector pGP‐L (SBI, Palo Alto, CA, USA). Lentivirus production was followed as standard protocol by transfecting 293T cells with recombinational shRNA vector, together with pVSVG‐I and pCMVΔR8.92 (Sigma, St. Louis, MO, USA). For cell transfection, thyroid cancer cells were cultured in 6‐well plates and transfected with the shTip60 or shCon with a multiplicity of infection of 60. After 48 h, the transfection efficiency was observed through a fluorescence microscope, and Tip60 expression was determined using western blot assay.

### Western blot analysis

Western blot assays were performed as described previously 21, 22. Briefly, total proteins were extracted from cells using 2× SDS sample buffer [100 mm Tris/HCl (pH 6.8), 10 mm EDTA, 4% SDS and 10% glycine]. Then, the protein concentration was measured with a bicinchoninic acid protein assay kit (Pierce Biotechnology, Rockford, IL, USA). Equal quantities of protein were separated by 12% SDS/polyacrylamide gel electrophoresis and transferred to polyvinylidene difluoride membranes (Millipore, Bedford, MA, USA). After blocking with 5% nonfat milk, the membranes were incubated with the primary antibody overnight at 4 °C. After washing with Tris‐buffered saline (TBS)/Tween‐20, the membranes were incubated with the horseradish peroxidase‐conjugated secondary antibody (1 : 5000; Santa Cruz Biotechnology, Santa Cruz, CA, USA) for 2 h at room temperature. Blots were visualized using an enhanced chemiluminescence kit (Pierce Biotechnology). The primary antibodies used were as follows: rabbit anti‐Tip60 (Abcam, Cambridge, MA, USA), rabbit anti‐ATM (Abcam), rabbit anti‐p‐ATM (Abcam), rabbit anti‐Acet (Cell Signaling Technology, Danvers, MA, USA), rabbit anti‐p‐Chk2 (Cell Signaling Technology) and rabbit anti‐p‐cdc25A (Cell Signaling Technology). Rabbit antiglyceraldehyde 3‐phosphate dehydrogenase (Proteintech, Rosemont, IL, USA) was used as the internal control. imagej software (National Institutes of Health, Bethesda, MD, USA) was used to measure the grey degree values of the bands.

### Immunoprecipitation assay

Cells were harvested and lysed using cold radioimmunoprecipitation assay lysis buffer for 30 min at 4 °C. After centrifugation at 14 000 ***g*** for 15 min at 4 °C, the supernatant was transferred to new tubes. A protein G‐agarose suspension was prepared, the agarose beads were washed with TBS, and a 50% protein A/G‐agarose solution was made with TBS. After again centrifuging at 14 000 ***g*** for 15 min at 4 °C, the supernatant was transferred to new tubes and the protein G‐agarose beads were discarded. Protein concentrations were measured via the bicinchoninic acid assay. Subsequently, the corresponding primary antibody was added into the total protein solution. Protein G‐agarose beads were added to capture the antigen–antibody complex, and the mixture was slowly shaken overnight at 4 °C. Then, the agarose beads–antigen–antibody complex was collected at 5000 ***g*** for 30 s and washed three times with precooled phosphate‐buffered saline. Finally, the supernatant was collected and analysed by the western blot assay. The experiment has been proceeded at least three independent times.

### 3‐(4,5‐Dimethylthiazol‐2‐yl)‐2,5‐diphenyltetrazolium bromide (MTT) assay

The MTT assay was performed as described previously 23. LNCaP and DU145 cells were infected with recombinant lentivirus (siCon or siTip60) for 96 h. The cells were then reseeded into 96‐well plates at a density of 2000 cells per well and treated with or without irradiation. After irradiated using X‐rays for 24, 48 and 72 h, 20 μL of the MTT solution (5 mg·mL^−1^) was added into each well. Following incubation for 4 h, 100 μL acidic isopropanol (10% SDS, 5% isopropanol and 10 mm HCI) was added to dissolve the formazan. Finally, the optical density of each well was measured using a microplate reader (Epoch, Biotek, Winooski, VT, USA) at a wavelength of 595 nm. The experiment has been proceeded at least three independent times.

### Statistical analysis

The experimental data were analysed using prism 5 software (GraphPad Software, La Jolla, CA, USA). The difference between two groups (siCon vs. siTip60) was determined using Student's *t*‐test. Data are shown as means ± standard deviation of three independent experiments. *P* < 0.05 was considered statistically significant.

## Results

### Tip60 was upregulated in radioresistant prostate cancer

**Figure 1:**
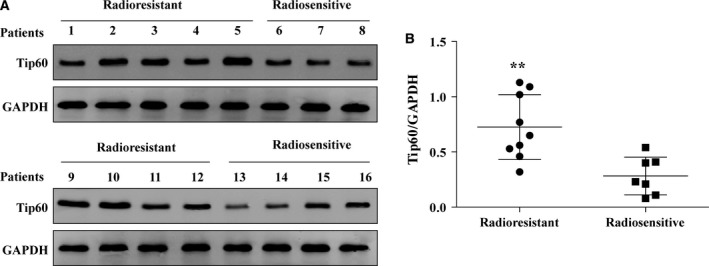
Tip60 is upregulated in radioresistant prostate cancer tissue. (A) The protein expression level of Tip60 in nine radioresistant and seven radiosensitive prostate cancer tissues was analysed by western blot. (B) Quantitative analysis of Tip60 protein in radioresistant and radiosensitive prostate cancer tissues has been provided. Student's *t* analysis indicated that Tip60 is upregulated in radioresistant prostate cancer tissue. **P < 0.01.

Tip60 protein expression was measured in radioresistant and radiosensitive prostate cancer tissues using western blot assays. Tip60 expression was significantly higher in the radioresistant than radiosensitive prostate cancer tissues (Fig. 1). This indicated that Tip60 may be closely associated with postoperative radiotherapy resistance of prostate cancer.

### X‐ray irradiation induced Tip60 expression and increased the acetylation of ATM in prostate cancer cells

**Figure 2:**
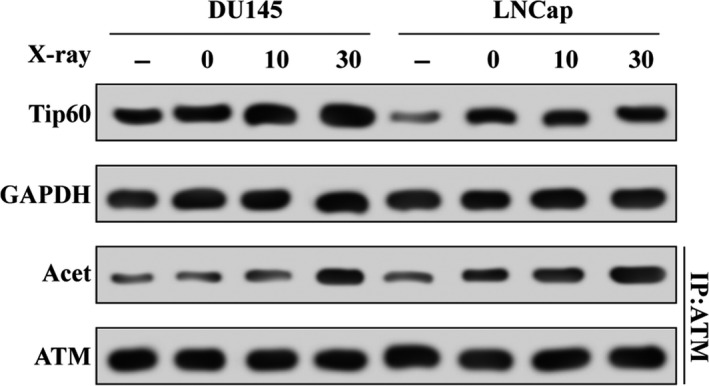
X‐ray irradiation induces Tip60 expression and increases the activation of ATM in prostate cancer cells. Tip60 protein expression and the acetylation (Acet) of ATM were analysed by western blot and immunoprecipitation (IP)–western blot assays in DU145 and LNCaP cells irradiated at a dose rate of 0.25 Gy·min^−1^ for 0, 10 or 30 min.

Next, two frequently used prostate cancer cell lines, DU145 and LNCaP, were irradiated with X‐rays at a dose of 0.25 Gy·min^−1^ for 0, 10 or 30 min. As shown in Fig. 2, the expression of Tip60 protein was increased in both cell lines after irradiation. Interestingly, we also found that the acetylation of ATM, a response element of Tip60‐dependent DNA damage, was upregulated time‐dependently.

### Depletion of Tip60 reduced the resistance of prostate cancer cells to radiation

**Figure 3:**
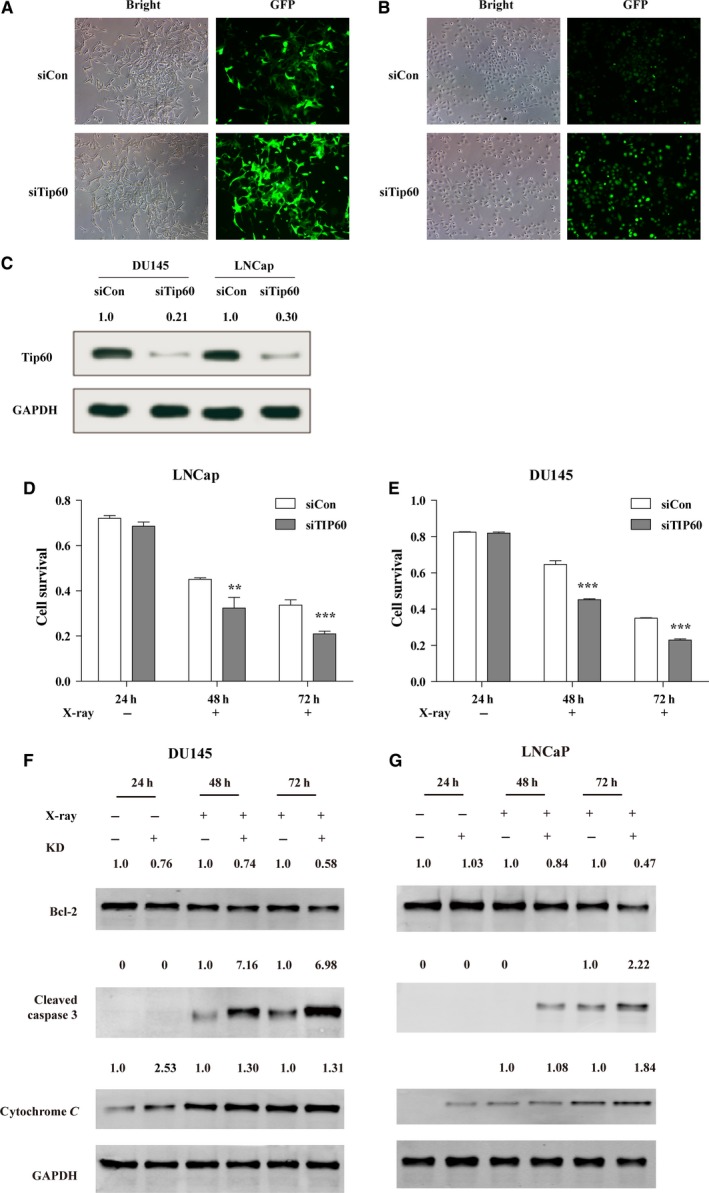
Depletion of Tip60 reduces the resistance of prostate cancer cells to radiation. (A) Representative bright and fluorescence images of LNCaP cells infected with control (siCon) and Tip60 (siTip60) small interfering RNA. (B) Representative bright and fluorescence images of DU145 cells infected with control (siCon) and Tip60 (siTip60) small interfering RNA. (C) Western blot analysis of the expression of Tip60 infected with control (siCon) and Tip60 (siTip60) small interfering RNA. (D) Survival of Tip60‐silenced LNCaP cells irradiated with 7.5 Gy X‐ray was measured by the MTT assay. (E) Survival of Tip60‐silenced DU145 cells irradiated with 7.5 Gy X‐ray was measured by the MTT assay. (F) Apoptosis‐relative biomarker expression pattern change in Tip60‐silenced DU145 cells irradiated with 7.5 Gy X‐ray. (G) Apoptosis‐relative biomarker expression pattern change in Tip60‐silenced LNCaP cells irradiated with 7.5 Gy X‐ray.

To further confirm whether Tip60 was involved in resistance to X‐ray irradiation in LNCaP and DU145 cells, both cells types were transfected with the siTip60 or siCon lentiviral vectors. Fluorescent images demonstrated that the transfection efficiencies of both siCon and siTip60 were above 90% in LNCaP and DU145 cells after 96 h of infection (Fig. 3A–C). Subsequently, the MTT assay was performed to detect the effects of Tip60 depletion on survival of LNCaP and DU145 cells that were irradiated with X‐rays. After X‐ray irradiation, depletion of Tip60 significantly inhibited the survival of LNCaP (Fig. 3D) and DU145 (Fig. 3E) cells at 48 and 72 h. In addition, we also determined apoptosis‐relative biomarkers, including Bcl‐2, cleaved caspase 3 and cytochrome C. The expression of Bcl‐2 was downregulated after X‐ray exposure in Tip60 KD group. Cleaved caspase 3 and cytochrome C showed a different trend (Fig. 3F,G). This indicated that depletion of endogenous Tip60 enhanced the sensitivity of prostate cancer cells to radiation *in vitro*.

### Tip60 regulated the sensitivity of prostate cancer cells to X‐ray irradiation via the ATM/Chk2/cdc25A pathway

**Figure 4:**
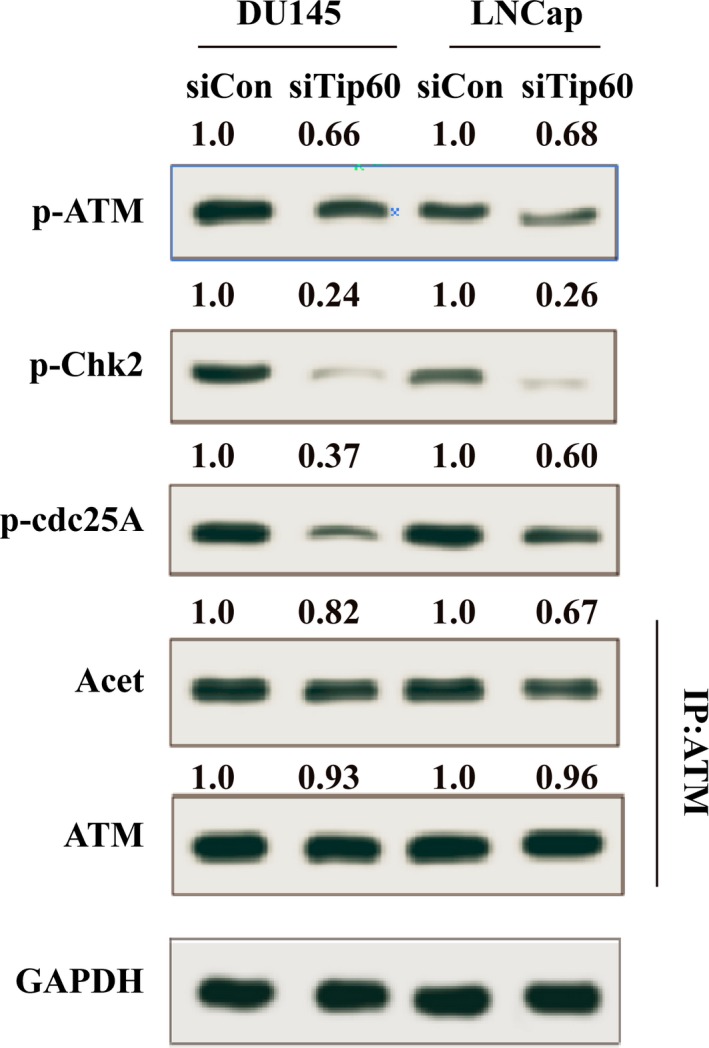
Tip60 regulates the sensitivity of prostate cancer cells to X‐ray irradiation via the ATM/Chk2/cdc25A pathway. Phosphorylation levels of AKT, Chk2 and cdc25A, and acetylation (Acet) of ATM, were analysed by western blot and immunoprecipitation (IP)–western blot assays in Tip60‐silenced DU145 and LNCaP cells irradiated at a dose of 7.5 Gy.

The molecular mechanism protecting human cells against radioresistant DNA synthesis mainly involves the ATM‐dependent pathway in osteosarcoma U2OS cells 24. Thus, we speculated that Tip60 could regulate activation of ATM in prostate cancer. As shown in Fig. 4, immunoprecipitation revealed that depletion of Tip60 significantly reduced the acetylation of ATM after X‐ray irradiation. Furthermore, the phosphorylation levels of molecules downstream of ATM, including Chk2, and cdc25A, were decreased in the siTip60 group in both DU145 and LNCaP cells after X‐ray irradiation. These results demonstrated that Tip60 may be involved in the resistance of prostate cancer cells to X‐ray irradiation, primarily through the ATM/Chk2/cdc25A pathway.

## Discussion

Prostate cancer is a complicated and polyfactorial disease. Clinical therapeutic strategies mainly include surgery, androgen deprivation therapy, radiotherapy or chemotherapy. Among these treatments, radiotherapy is complementary for surgical patients and is also an available therapeutic approach for unrespectable prostate neoplasms 25. However, a portion of treated prostate cancer patients develop resistance to radiation during therapy. This results in therapeutic failure and poor prognosis. Thus, resistance to radiotherapy is an urgent problem in the current clinical situation.

Accumulating reports have focused on the mechanisms and molecular markers of radiation resistance 26, 27. Our study is the first to show that the resistance of prostate cancer to X‐ray irradiation correlates with the expression of Tip60. Furthermore, Tip60 may modulate the survival of irradiated prostate cancer DU145 and LNCaP cells via activating the ATM/Chk2/cdc25A pathway.

It is well known that X‐ray irradiation can injure cells by fracturing DNA or the generation of free radicals that cause DNA damage 28. Extensive evidence suggests that the function of Tip60 not only is reflected in transcriptional regulation but also involves numerous other processes including signal transduction, DNA damage repair and the regulation of cell cycle checkpoints and apoptosis through the acetylation of its histone 29. Tip60 plays a direct role in activating ATM 30. During DNA damage, ATM activation relies on its rapid acetylation induced by Tip60. Furthermore, overexpression of a Tip60 HAT mutant or silencing of Tip60 restrains ATM acetylation and Chk2 phosphorylation that are dependent on the ATM kinase 30, 31. Moreover, Chk2 phosphorylation could activate cyclin‐dependent kinase 2, which is diminished in response to DNA damage 24, 32.

Other studies also demonstrated that ATM activation was often connected with DNA double‐strand breaks caused by ionizing radiation or radiomimetic drugs 33. Additionally, Busino *et al*. 34 established that cdc25A‐ and cyclin‐dependent kinase 2 modulated the transition between the G1/S and G2/M phases of the cell cycle and were also involved in ionizing radiation‐activated checkpoint pathways. In our study, we found that the Tip60 protein level was much higher in radioresistant than radiosensitive prostate cancer tissues. We also found increased ATM acetylation in response to X‐ray irradiation in DU145 and LNCaP prostate cancer cells. Moreover, after X‐ray irradiation, Tip60 knockdown significantly decreased the survival of prostate cancer cells, reduced the acetylation of ATM and decreased the phosphorylation levels of AKT, Chk2 and cdc25A. However, animal experiments are needed to explore the effect of Tip60 knockdown on the radioresistance of prostate cancer *in vivo*.

## Conclusions

In this study, knockdown of Tip60 could enhance the sensitivity of prostate cancer cells to X‐ray irradiation by inducing apoptosis, confirmed by the expression pattern change of Bcl‐2, cleaved caspase 3 and cytochrome C. In addition, we also found depletion of Tip60 significantly reduced the acetylation of ATM after X‐ray irradiation and a further study demonstrated the phosphorylation levels of molecules downstream of ATM, including Chk2, and cdc25A, were decreased after Tip60 knockdown. These findings suggest that Tip60 could serve as a promising predictive factor for prostate cancer patients receiving radiotherapy. Our results also may contribute to implementing individualized therapeutic regimens for prostate cancer patients with dysregulated Tip60 expression, thereby improving the efficacy of radiotherapy.

## Author contributions

WH designed experiments and improved the manuscript. XX, ZX and CW performed experiments and analysed data. CF and JZ coordinated the studies and improved the manuscript. LX and XQ drafted the manuscript. JD, FS and DX participated in data analysis. All authors read and approved the manuscript.
